# A comparison of five epidemiological models for transmission of SARS-CoV-2 in India

**DOI:** 10.1186/s12879-021-06077-9

**Published:** 2021-06-07

**Authors:** Soumik Purkayastha, Rupam Bhattacharyya, Ritwik Bhaduri, Ritoban Kundu, Xuelin Gu, Maxwell Salvatore, Debashree Ray, Swapnil Mishra, Bhramar Mukherjee

**Affiliations:** 1 Department of Biostatistics, University of Michigan, Ann Arbor, MI 48109 USA; 2 Indian Statistical Institute, Kolkata, West Bengal 700108 India; 3 Center for Precision Health Data Science, University of Michigan, Ann Arbor, MI 48109 USA; 4 Department of Epidemiology, University of Michigan, Ann Arbor, MI 48109 USA; 5 Department of Epidemiology, Johns Hopkins Bloomberg School of Public Health, Baltimore, MD 21205 USA; 6 Department of Biostatistics, Johns Hopkins Bloomberg School of Public Health, Baltimore, MD 21205 USA; 7 School of Public Health, Imperial College London, London, W2 1PG UK

**Keywords:** Compartmental models, Low and middle income countries, Prediction uncertainty, Statistical models

## Abstract

**Background:**

Many popular disease transmission models have helped nations respond to the COVID-19 pandemic by informing decisions about pandemic planning, resource allocation, implementation of social distancing measures, lockdowns, and other non-pharmaceutical interventions. We study how five epidemiological models forecast and assess the course of the pandemic in India: a baseline curve-fitting model, an extended SIR (eSIR) model, two extended SEIR (SAPHIRE and SEIR-fansy) models, and a semi-mechanistic Bayesian hierarchical model (ICM).

**Methods:**

Using COVID-19 case-recovery-death count data reported in India from March 15 to October 15 to train the models, we generate predictions from each of the five models from October 16 to December 31. To compare prediction accuracy with respect to reported cumulative and active case counts and reported cumulative death counts, we compute the symmetric mean absolute prediction error (SMAPE) for each of the five models. For reported cumulative cases and deaths, we compute Pearson’s and Lin’s correlation coefficients to investigate how well the projected and observed reported counts agree. We also present underreporting factors when available, and comment on uncertainty of projections from each model.

**Results:**

For active case counts, SMAPE values are 35.14% (SEIR-fansy) and 37.96% (eSIR). For cumulative case counts, SMAPE values are 6.89% (baseline), 6.59% (eSIR), 2.25% (SAPHIRE) and 2.29% (SEIR-fansy). For cumulative death counts, the SMAPE values are 4.74% (SEIR-fansy), 8.94% (eSIR) and 0.77% (ICM). Three models (SAPHIRE, SEIR-fansy and ICM) return total (sum of reported and unreported) cumulative case counts as well. We compute underreporting factors as of October 31 and note that for cumulative cases, the SEIR-fansy model yields an underreporting factor of 7.25 and ICM model yields 4.54 for the same quantity. For total (sum of reported and unreported) cumulative deaths the SEIR-fansy model reports an underreporting factor of 2.97. On October 31, we observe 8.18 million cumulative reported cases, while the projections (in millions) from the baseline model are 8.71 (95% credible interval: 8.63–8.80), while eSIR yields 8.35 (7.19–9.60), SAPHIRE returns 8.17 (7.90–8.52) and SEIR-fansy projects 8.51 (8.18–8.85) million cases. Cumulative case projections from the eSIR model have the highest uncertainty in terms of width of 95% credible intervals, followed by those from SAPHIRE, the baseline model and finally SEIR-fansy.

**Conclusions:**

In this comparative paper, we describe five different models used to study the transmission dynamics of the SARS-Cov-2 virus in India. While simulation studies are the only gold standard way to compare the accuracy of the models, here we were uniquely poised to compare the projected case-counts against observed data on a test period. The largest variability across models is observed in predicting the “total” number of infections including reported and unreported cases (on which we have no validation data). The degree of under-reporting has been a major concern in India and is characterized in this report. Overall, the SEIR-fansy model appeared to be a good choice with publicly available R-package and desired flexibility plus accuracy.

**Supplementary Information:**

The online version contains supplementary material available at 10.1186/s12879-021-06077-9.

## Background

Coronavirus disease 2019 (COVID-19) is an infectious disease caused by severe acute respiratory syndrome coronavirus 2 (SARS-CoV-2) [1]. At the time of revising this paper (March 24, 2021), roughly 124 million cases have been reported worldwide. The disease was first identified in Wuhan, Hubei Province, China in December 2019 [2]. Since then, more than 2.74 million lives have been lost as a direct consequence of the disease. Notable outbreaks were recorded in the United States of America, Brazil and India -- which remains a crucial battleground against the outbreak. The Indian government imposed very strict lockdown measures early in the course of the pandemic in order to reduce the spread of the virus. Said measures have not been as effective as was intended [3], with India now reporting the largest number of confirmed cases in Asia, and the third highest number of confirmed cases in the world after the United States and Brazil [4], with the number of confirmed cases crossing the 10 million mark on December 18, 2020. On March 24, 2020, the Government of India ordered a 21-day nationwide lockdown, later extending it until May 3. This was followed by two-week extensions starting May 3 and 17 with substantial relaxations. From June 1, the government started ‘unlocking’ most regions of the country in five unlock phases. In order to formulate and implement policy geared toward containment and mitigation, it is important to recognize the presence of highly variable contagion patterns across different Indian states [5]. India saw a decay in the virus curve in September, 2020 with daily number of cases going below 10,000. At the time of revising the paper, the daily incidence curve is sharply rising again, as India faces its second wave. There is a rising interest in studying potential trajectories that the infection can take in India to improve policy decisions.

A spectrum of models for projecting infectious disease spread have become widely popular in wake of the pandemic. Some popular models include the ones developed at the Institute of Health Metrics (IHME) [6] (University of Washington, Seattle) and at the Imperial College London [7]. The IHME COVID-19 project initially relied on an extendable nonlinear mixed effects model for fitting parametrized curves to COVID-19 data, before moving to a compartmental model to analyze the pandemic and generate projections. The Imperial College model (henceforth referred to as ICM) works backwards from observed death counts to estimate transmission that occurred several weeks ago, allowing for the time lag between infection and death. A Bayesian mechanistic model is introduced - linking the infection cycle to observed deaths, inferring the total population infected (attack rates) as well as the time-varying reproduction number *R*(*t*). With the onset of the pandemic, there has been renewed interest in multi-compartment models, which have played a central role in modeling infectious disease dynamics since the twentieth century [8]. The simplest of compartmental models include the standard SIR [9] model, which has been extended [10] to incorporate various types of time-varying quarantine protocols, including government-level macro isolation policies and community-level micro inspection measures. Further extensions include one which adds a spatial component to this temporal model by making use of a cellular automata structure [11]. Larger compartmental models include those which incorporate different states of transition between susceptible, exposed, infected and removed (SEIR) compartments, which have been used in the early days of the pandemic in the Wuhan province of China [12]. The SEIR compartmental model has been further extended to the SAPHIRE model [13], which accounts for the infectiousness of asymptomatic [14] and pre-symptomatic [15] individuals in the population (both of which are crucial transmission features of COVID-19), time varying ascertainment rates, transmission rates and population movement.

Researchers and policymakers are relying on these models to plan and implement public health policies at the national and local levels. New models are emerging rapidly. Models often have conflicting messages, and it is hard to distinguish a good model from an unreliable one. Different models operate under different assumptions and provide different deliverables. In light of this, it is important to investigate and compare the findings of various models on a given test dataset. While some work has been done in terms of trying to reconcile results from different models of disease transmission that can be fit to emerging data [16], more comparisons need to be done to investigate how differences between competing models might lead to differing projections on the same dataset. In the context of India, such head-to-head comparison across models are largely unavailable.

We consider five different models of different genre, starting from the simplest baseline model. The baseline model we investigate relies on curve-fitting methods, with cumulative number of infected cases modeled as an exponential process [17]. Next, we consider the extended SIR (eSIR) model [10], which uses a Bayesian hierarchical model to generate projections of proportions of infected and removed people at future time points. The SAPHIRE [13] model has been demonstrated to reconstruct the full-spectrum dynamics of COVID-19 in Wuhan between January and March 2020 across five periods defined by events and interventions. Using this, we study the evolution of the pandemic in India over nine well-defined lockdown and unlock periods, each with distinct transmission and ascertainment features. Another model, SEIR-fansy [18] modifies the SEIR model to account for high false negative rate and symptom-based administration of COVID-19 tests. Finally, we study the ICM model, which utilizes a semi-mechanistic Bayesian hierarchical model based on renewal equations that model infections as a latent process and links deaths to infections with the help of survival analysis*.* Each of the models mentioned above have had appreciable success in being able to satisfactorily analyze and project the trajectory of the pandemic in different countries [19–21].

In order to fairly compare and contrast the models mentioned above, we study their respective treatment of the different lockdown and unlock periods declared by the Government of India. Additionally, we compare their projections based on reported data, with special emphasis on how the models deal with (if they do, at all) under-reporting and under-detection of COVID-cases, which has been a major point of discussion in the scientific community, particularly for India [22]. We also compare the uncertainty associated with the projections across the models which is often overlooked in the literature.

The rest of the paper is organized as follows. In *Section 2* we provide an overview of the various models considered in our analysis. The supplement has detailed discussion on the formulation, assumptions and estimation methods utilized by each of the models. We present the numerical findings of our comparative investigation of the models in *Section 3* by comparing projected COVID-counts (i.e., case and death counts associated with COVID-19) and (wherever possible) parameter estimates which help understand transmission dynamics of the pandemic. Next, in *Section 4* we discuss sensitivity analyses and note applications of the models studied in the context of data from countries other than India. Finally, we discuss the implications of our findings in *Section 5*.

## Methods

### Overview of models

In this section, we discuss the assumptions and formulation of each of the five classes of models described above. Table 1 provides an overview of the models compared in this article.

**Table 1:** Overview of models studied

Name of model	Comments	Input(s) and output(s)	Parameter(s) and estimation
Baseline (Bhardwaj, R. 2020) [17]	Curve-fitting model. Cumulative number of infected cases modeled as exponential process, with growth rate *λ*.	Daily time series of number of infected individuals from *T*_0_ till *T*_1_^1^ (as input) and from *T*_1_ to *T*_2_^2^ (as output).	Time varying growth rate of infection is estimated from input and modeled using least-squares regression. Estimation involves implementing MCMC^3^ methods for a Bayesian framework.
eSIR (Wang, L. et al., 2020) [23–25]	Extension of the standard SIR^2^ compartmental model.	Daily time series data on proportion of infected and recovered individuals from *T*_0_ till *T*_1_^1^ (as input) and from *T*_1_ to *T*_2_^2^ along with posterior distribution of parameters and prevalence values of the three compartments in the model (as output).	*β* and *γ* control transmission and removal rates respectively. ***λ*** and *κ* control variability of observed and latent processes respectively. Estimation involves implementing MCMC^3^ methods for a hierarchical Bayesian framework.
SAPHIRE (Hao, X. et al., 2020) [13]	Extension of the standard SEIR^2^ compartmental model.	Daily time series data from *T*_0_ till *T*_1_^1^ on count of infected individuals (as input) and count of infected and removed individuals from *T*_1_ to *T*_2_^2^ along with posterior distributions of parameters (as output). Unreported cases are also presented.	See Section 2.1.c for details on parameters. Estimation involves implementing MCMC^3^ methods for a Bayesian framework.
SEIR-*fansy* (Bhaduri, R., Kundu, R. et al., 2020) [18, 26]	Another extension of standard SEIR^2^, accounting for the possible effect of misclassifications due to imperfect testing.	Daily time series data from *T*_0_ till *T*_1_^1^ on proportion of dead, infected and recovered individuals (as input) and from *T*_1_ to *T*_2_^2^ along with posterior distributions of parameters and prevalence values of compartments in the model (as output). Unreported cases and deaths are also projected.	See ***Supplementary Table S******1*** for details on parameters. Estimation involves implementing MCMC^3^ methods for a hierarchical Bayesian framework.
ICM (Flaxman et.al., 2020) [7]	Renewal equation used to model infections as a latent process. Deaths are linked to infections via a survival distribution. Accounts for changes in behavior and various governmental policies enacted.	Daily time series data from *T*_0_ till *T*_1_^1^ on count of dead individuals (as input) and from *T*_1_ to *T*_2_ (as output). Posterior over infections, deaths and various parameters. Infections include both symptomatic and asymptomatic ones.	See Section 2.1.e for details on parameters. Estimation is done via HMC^4^ using STAN.

^1^ *T*_0_: time of crossing 50 confirmed cases – March 12, 2020. *T*_1_: October 15, 2020. *T*_2_: December 31, 2020

^2^ *S*(*E*)*IR* susceptible-(exposed)-infected-removed

^3^ *MCMC* Markov chain-Monte Carlo

^4^ Hamiltonian Monte Carlo

#### Baseline model

##### Overview

**Fig. 1:**
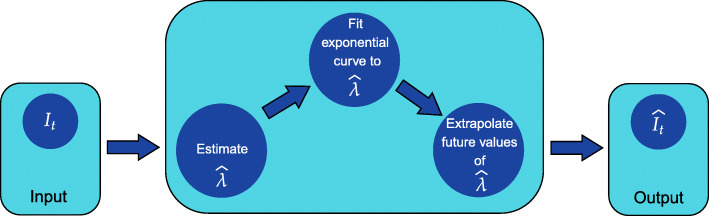
Schematic overview of the baseline model

The baseline model we investigate aims to predict the evolution of the COVID-19 pandemic by means of a regression-based predictive model [17]. More specifically, the model relies on a regression analysis of the daily cumulative count of infected cases based on the least-squares fitting. In particular, the growth rate of the infection is modeled as an exponentially decaying process. Figure 1 provides a schematic overview of this model.

##### Formulation

The baseline model assumes that the following simple differential equation governs the evolution of a disease in a fixed population:

1 $$ \frac{dI(t)}{dt}=\uplambda I(t) $$

where *I*(*t*) is defined as the number of infected people at time *t* and λ is the growth rate of infection. Unlike the other models described in subsequent sections, the baseline model analyses and projects only the cumulative number of infections, and not counts/proportions associated with other compartments like deaths and recoveries. The model uses reported field data of the infections in India over a specific time period. The growth rate can be numerically approximated from Eq. (1) above as

2 $$ \hat{\uplambda_{\mathrm{t}}}=\frac{I_t-{I}_{t-2}}{2\cdotp {I}_t} $$

Having estimated the growth rate, the model uses a least-squares method to fit an exponential time-varying curve to $\hat{\uplambda_t}$, obtained from Eq. (2) above. Since all the other methods involve Bayesian estimation methods and use posterior distributions to obtain estimates and associated credible intervals, we place a non-informative prior on the random error in the above curve fitting method [27] to ensure comparable results. Specifically, we consider a uniform prior for the log of error variance. Using projected values of $\hat{\lambda_t},$ we extrapolate the number of infections which will occur in future. The baseline model described above has been implemented in R [28] using standard packages for exponential curve fitting.

#### Extended SIR (eSIR) model

##### Overview

**Fig. 2:**
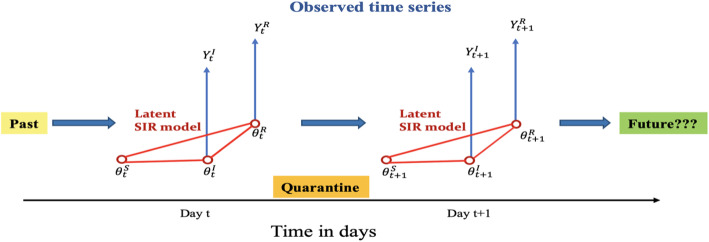
The eSIR model with a latent SIR model on the unobserved proportions. Reproduced from Wang et al., 2020 [10]

We use an extension of the standard susceptible-infected-removed (SIR) compartmental model known as the extended SIR (eSIR) model [10]. To implement the eSIR model, a Bayesian hierarchical framework is used to model time series data on the proportion of individuals in the infected and removed compartments. Markov chain Monte Carlo (MCMC) methods are used to implement this model, which provides not only posterior estimation of parameters and prevalence values associated with all three compartments of the SIR model, but also predicted proportions of the infected and the removed people at future time points. Figure 2 is a diagrammatic representation of the eSIR model.

##### Formulation

The eSIR model assumes the true underlying probabilities of the three compartments follow a latent Markov transition process and require observed daily proportions of infected and removed cases as input.

The observed proportions of infected and removed cases on day *t* are denoted by ${Y}_t^I$ and ${Y}_t^R$, respectively. Further, we denote the true underlying probabilities of the S, I, and R compartments on day *t* by ${\theta}_t^S$, ${\theta}_t^I$, and ${\theta}_t^R$, respectively, and assume that for any *t*, ${\theta}_t^S+{\theta}_t^I+{\theta}_t^R=1$. Assuming a usual SIR model on the true proportions we have the following set of differential equations

3a $$ \frac{d{\theta}_t^S}{dt}=-\beta {\theta}_t^S{\theta}_t^I, $$

3b $$ \frac{d{\theta}_t^I}{dt}=\beta {\theta}_t^S{\theta}_t^I-\gamma {\theta}_t^I, $$

3c $$ \frac{d{\theta}_t^R}{dt}=\gamma {\theta}_t^I, $$

where *β* > 0 denotes the disease transmission rate, and *γ* > 0 denotes the removal rate. The basic reproduction number *R*_0_ ≔ *β*/*γ* indicates the expected number of cases generated by one infected case in the absence of any intervention and assuming that the whole population is susceptible. We assume a Beta-Dirichlet state space model for the observed infected and removed proportions, which are conditionally independently distributed as

4a $$ {Y}_t^I\mid {\boldsymbol{\theta}}_{\boldsymbol{t}},\boldsymbol{\tau} \sim Beta\left({\lambda}^I{\theta}_t^I,{\lambda}^I\left(1-{\theta}_t^I\right)\right) $$

4b $$ {Y}_t^R\mid {\boldsymbol{\theta}}_{\boldsymbol{t}},\boldsymbol{\tau} \sim Beta\left({\lambda}^R{\theta}_t^R,{\lambda}^R\left(1-{\theta}_t^R\right)\right). $$

Further, the Markov process associated with the latent proportions is built as:

5 $$ {\boldsymbol{\theta}}_{\boldsymbol{t}}\mid {\boldsymbol{\theta}}_{\boldsymbol{t}-\mathbf{1}},\boldsymbol{\tau} \sim \mathrm{D} irichlet\left(\kappa f\left({\boldsymbol{\theta}}_{\boldsymbol{t}-\mathbf{1}},\beta, \gamma \right)\right) $$

where ***θ***_***t***_ denotes the vector of the underlying population probabilities of the three compartments, whose mean is modeled as an unknown function of the probability vector from the previous time point, along with the transition parameters. $\boldsymbol{\tau} =\left(\beta, \gamma, {\boldsymbol{\theta}}_{\mathbf{0}}^T,\boldsymbol{\lambda}, \kappa \right)$ denotes the whole set of parameters where *λ*^*I*^, *λ*^*R*^ and *κ* are parameters controlling variability of the observation and latent process, respectively. The function *f*(·) is then solved as the mean transition probability determined by the SIR dynamic system, using a fourth order Runge-Kutta approximation [29].

##### Priors and MCMC algorithm

The prior on the initial vector of latent probabilities is set as ${\boldsymbol{\theta}}_{\mathbf{0}}\sim \mathrm{Dirichlet}\left(1-{Y}_1^I-{Y}_1^R,{Y}_1^I,{Y}_1^R\right)$, ${\theta}_0^S=1-{\theta}_0^I-{\theta}_0^R$. The prior distribution of the basic reproduction number is lognormal such that *E*(*R*_0_) = 3.28 [30] (this value was also confirmed by calculating the average time-varying R(t) by from January 30 till March 24, 2020, using the package developed by [31]). The prior distribution of the removal rate is also lognormal such that *E*(*γ*) = 0.5436. We use the proportion of death within the removed compartment as 0.0184 so that the initial infection fatality ratio is 0.01 [32]. For the variability parameters, the default choice is to set large variances in both observed and latent processes, which may be adjusted over the course of epidemic with more data becoming available: $\kappa, {\lambda}^I,{\lambda}^R\ \overset{iid}{\sim }\ \mathrm{Gamma}\left(2,{10}^{-4}\right).$

Denoting *t*_0_ as the last date of data availability, and assuming that the forecast spans over the period [*t*_0_ + 1, *T*], the eSIR algorithm is as follows.

- Step 0. Take *M* draws from the posterior $\left[{\boldsymbol{\theta}}_{\mathbf{1}:{\boldsymbol{t}}_{\mathbf{0}}},\boldsymbol{\tau} |{\boldsymbol{Y}}_{\mathbf{1}:{\boldsymbol{t}}_{\mathbf{0}}}\right]$.
- Step 1. For each solution path *m* ∈ {1, …, *M*}, iterate between the following two steps via MCMC.
  i. Draw $$ {\boldsymbol{\theta}}_{\boldsymbol{t}}^{\left(\boldsymbol{m}\right)} $$ from $$ \left[\left.{\boldsymbol{\theta}}_{\boldsymbol{t}}\right|{\boldsymbol{\theta}}_{t-1}^{\left(m-1\right)},{\boldsymbol{\tau}}^{(m)}\right],t\in \left\{{t}_0+1,\dots, T\right\} $$.
  ii. Draw ${\boldsymbol{Y}}_{\boldsymbol{t}}^{\left(\boldsymbol{m}\right)}$ from $\left[\left.{\boldsymbol{Y}}_{\boldsymbol{t}}\right|{\boldsymbol{\theta}}_t^{(m)},{\boldsymbol{\tau}}^{(m)}\right],t\in \left\{{t}_0+1,\dots, T\right\}$.

##### Implementation

We implement the proposed algorithm in R package *rjags* [33] and the differential equations were solved via the fourth-order Runge–Kutta approximation. To ensure the quality of the MCMC procedure, we fix the adaptation number (which denotes the number of MCMC samples discarded by JAGS in order to tune parameters which in turn improves speed or de-correlation of sampling) at 10^4^, thin the chain by keeping one draw from every 10 random draws to further reduce autocorrelation, set a burn-in period of 10^5^ draws under 2 × 10^5^ iterations for four parallel chains. This implementation provides not only posterior estimation of parameters and prevalence of all the three compartments in the SIR model, but also predicts proportions of the infected and the removed people at future time point(s). The R package for implementing this general model for understanding disease dynamics is publicly available at *https://github.com/lilywang1988/eSIR**.*

#### SAPHIRE model

##### Overview

**Fig. 3:**
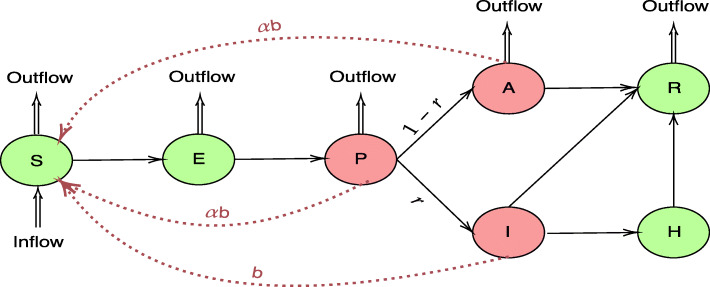
The SAPHIRE model includes seven compartments: susceptible (S), exposed (E), pre-symptomatic infectious (P), reported infectious (I), unreported infectious (A), isolation in hospital (H) and removed (R)

This model [13] extends the classic SEIR model to estimate COVID-related transmission parameters, in addition to projecting COVID-19 case counts, while accounting for pre-symptomatic infectiousness, time-varying ascertainment rates (i.e. reporting rates), transmission rates and population movements. Figure 3 provides a schematic diagram of the compartments and transitions conceptualized in this model. The model includes seven compartments: susceptible (S), exposed (E), pre-symptomatic infectious (P), reported infectious (I), unreported infectious (A), isolation in hospital (H) and removed (R). Compared with the classic SEIR model, SAPHIRE explicitly models population movement and introduce two additional compartments (A and H) to account for the fact that only reported cases would seek medical care and thus be quarantined by hospitalization. The model described and implemented here relies on the same methodology and arguments as presented by [13]. The only difference is that while the original model analyzed data from China over a time period of December 2019 to March 2020 (which constituted the initial days of the pandemic in China), we analyze data from India. Additionally, the original manuscript adjusted the model to account for population movement. Data on population movement not being available consistently over time and regions in India, we make no such modifications. We further note that the SAPHIRE model returns reported and unreported cumulative COVID-case counts, in addition to cumulative counts of the removed compartment. As such, for the purpose of comparisons, the SAPHIRE model is used only to study cumulative COVID-case counts (reported and unreported). The R package for implementing this general model for understanding disease dynamics is publicly available at *https://github.com/chaolongwang/SAPHIRE**.*

##### Formulation

The dynamics of the 7 compartments described above at time *t* are described by the set of ordinary differential equations

6a $$ \frac{dS}{dt}=n-\frac{bS\left(\alpha P+\alpha A+I\right)}{N}-\frac{nS}{N}, $$

6b $$ \frac{dE}{dt}=\frac{bS\left(\alpha P+\upalpha A+I\right)}{N}-\frac{E}{D_e}-\frac{nE}{N}, $$

6c $$ \frac{dP}{dt}=\frac{E}{D_e}-\frac{P}{D_P}-\frac{nP}{N}, $$

6d $$ \frac{dA}{dt}=\frac{\left(1-r\right)P}{D_P}-\frac{A}{D_i}-\frac{nA}{N}, $$

6e $$ \frac{dI}{dt}=\frac{rP}{D_P}-\frac{I}{D_i}-\frac{I}{D_q}, $$

6f $$ \frac{dH}{dt}=\frac{I}{D_q}-\frac{H}{D_h}, $$

6g $$ \frac{dR}{dt}=\frac{A+I}{D_i}+\frac{H}{D_h}-\frac{nR}{N}, $$

in which *b* is the transmission rate for reported cases (defined as the number of individuals that an reported case can infect per day), α is the ratio of the transmission rate of unreported cases to that of reported cases, *r* is the ascertainment rate, *D*_*e*_ is the latent period, *D*_*p*_ is the pre-symptomatic infectious period, *D*_*i*_ is the symptomatic infectiousness period, *D*_*q*_ is the duration from illness onset to isolation and *D*_*h*_ is the isolation period in the hospital. Further, we set *N* = 1.34 × 10^9^ as the population size for India and set *n* = 0 to indicate no incoming or outgoing travelers.

Under this setup, the reproductive number *R* (as presented in the original manuscript) may be expressed as

7 $$ R=\alpha b{\left({D}_{\mathrm{p}}^{-1}+\frac{n}{N}\right)}^{-1}+\left(1-r\right)\alpha b{\left({D}_{\mathrm{i}}^{-1}+\frac{n}{N}\right)}^{-1}+ rb{\left({D}_{\mathrm{i}}^{-1}+{D}_{\mathrm{q}}^{-1}\right)}^{-1}, $$

in which the three terms represent infections contributed by pre-symptomatic individuals, unreported cases and reported cases, respectively. The model adjusts the infectious periods of each type of case by taking isolation of patients who test positive $\left(\mathrm{by}\ \mathrm{means}\ \mathrm{of}\ {D}_q^{-1}\right)$ into account.

##### Initial states and parameter settings

We set α = 0.55, assuming lower transmissibility for unreported cases [34]. Compartment *P* contains both reported and unreported cases in the pre-symptomatic phase. We set the transmissibility of *P* to be the same as unreported cases, because it has previously been reported that the majority of cases are unreported [34]. We assume an incubation period of 5.2 days and a pre-symptomatic infectious period *D*_*p*_ = 2.3 days [35, 36]. The latent period was *D*_*e*_ = 2.9 days. Since pre-symptomatic infectiousness was estimated to account for 44% of the total infections from reported cases [35], we set the mean of total infectious period as (*D*_*p*_ + *D*_*i*_) = *D*_*p*_/0.44 = 5.2 days, assuming constant infectiousness across the pre-symptomatic and symptomatic phases of reported cases [37] – thus the mean symptomatic infectious period was *D*_*i*_ = 2.9 days. We set a long isolation period of *D*_*h*_ = 17 days, based on a study investigating hospitalisation of COVID-19 patients in the state of Karnataka [38]. The duration from the onset of symptoms to isolation was estimated to be *D*_*q*_ = 7 [23, 39] as the median time length from onset to confirmed diagnosis. On the basis of the parameter settings above, the initial state of the model is specified on March 15. The initial number of reported symptomatic cases *I*(0) is specified as the number of reported cases who experienced symptom onset during 12–14 March. The initial ascertainment rate is assumed to be *r*_0_ = 0.10 [40], and thus the initial number of unreported cases is $A(0)={r}_0^{-1}\left(1-{r}_0\right)I(0)$. *P*_1_(0) and *E*_1_(0) denote the numbers of reported cases in which individuals experienced symptom onset during 15–16 March and 17–19 March, respectively. Then, the initial numbers of exposed and pre-symptomatic individuals are set as $E(0)={r}_0^{-1}{E}_1(0)$ and $P(0)={r}_0^{-1}{P}_1(0)$, respectively. The initial number of the hospitalized cases *H*(0) is set as half of the cumulative reported cases on 8 March since *D*_*q*_ = 7 and there would be more severe cases among the reported cases in the early phase of the epidemic.

##### Likelihood and MCMC algorithm

Considering the time-varying strength of control measures implemented in India over the trajectory of the pandemic, we chose to break the training period into ten sequential blocks: pre-lockdown (March 15–24), lockdown phases 1, 2, 3, and 4 (March 25 – April 14, April 15 – May 3, May 4–17, and May 18–31 respectively) followed by unlock phases 1, 2, 3, 4 and 5 (June 1–30, July 1–31, August 1–31, September 1–30 and October 1–15 respectively). In other words, the model assumes that the value of *b* (and *r*) corresponding to the *i*^*th*^ lockdown period to vary as *b*_*i*_(and *r*_*i*_) for *i* = 1, 2, 3, …, 10. The observed number of reported cases in which individuals experience symptom onset on day *t* – denoted by *x*_*t*_ – is assumed to follow a Poisson distribution with rate ${\uplambda}_t=r{P}_{t-1}{D}_p^{-1}$, with *P*_*t*_ denoting the expected number of pre-symptomatic individuals on day *t*. The following likelihood equation is used to fit the model using observed data from March 15 (*T*_0_) to October 15 (*T*_1_).

$$ L\left({b}_1,{b}_2,\dots, {b}_{10},{r}_1,{r}_2,\dots, {r}_{10}\right)=\prod \limits_{t={T}_0}^{T_1}\frac{{\mathrm{e}}^{-{\uplambda}_{\mathrm{t}}}{\lambda}_t^{x_t}}{x_t!}, $$

and the model is used to predict COVID-counts from October 16 to December 31. A non-informative prior of *U*(0, 2) is used for *b*_1_, *b*_2_, …, *b*_10_. For *r*_1_, an informative prior of Beta(10, 90) is used based on the findings of [40]. We reparameterise *r*_2_, …, *r*_10_ as

$$ \mathrm{logit}\left({r}_i\right)=\mathrm{logit}\left({r}_{i-1}\right)+{\updelta}_i\ \mathrm{for}\ i=2,3,\dots, 10 $$

where logit(*t*) = log(*t*/(1 − *t*)) is the standard logit function. In the MCMC, δ_*i*_ ∼ *N*(0, 1) for *i* = 2, 3, …, 10. A burn-in period of 100,000 iterations is fixed, with a total of 200,000 iterations being run.

#### SEIR-fansy model

##### Overview

One of the problems with applying a standard SIR model in the context of the COVID-19 pandemic is the presence of a long incubation period. As a result, extensions of SIR model like the SEIR model are more applicable. In the previous subsection, we have seen an extension which includes the ‘pre-symptomatic infectious’ compartment (people who are infected at time t and contributing to the spread of the virus, but do not show any symptom yet). In the SEIR-fansy model, we use an alternate formulation by defining an ‘untested infectious’ compartment for infected people who are spreading infection but are not tested after the incubation period. This compartment is necessary because there is a large proportion of infected people who are not being tested (a part of them are asymptomatic or mildly symptomatic but for a country like India there are other reasons like access to care and stigma that can prevent someone from getting tested/diagnosed). We have assumed that after the ‘exposed’ compartment, a person enters either the ‘untested infectious’ compartment or the ‘tested infectious’ compartment. To incorporate the possible effect of misclassifications due to imperfect testing, we include a compartment for false negatives (infected people who are tested but reported as negative). As a result, after being tested, an infected person enters either into the ‘false negative’ compartment or the ‘tested positive’ compartment (infected people who are tested and reported to be positive). We keep separate compartments for the recovered and deceased persons coming from the untested and false negatives compartments which are ‘recovered unreported’ and ‘deceased unreported’ respectively. For the ‘tested positive’ compartment, the recovered and the death compartments are denoted by ‘recovered reported’ and ‘deceased reported’ respectively. Thus, we divide the entire population into ten main compartments: S (Susceptible), E (Exposed), T (Tested), U (Untested), P (Tested positive), F (Tested False Negative), RR (Reported Recovered), RU (Unreported Recovered), DR (Reported Deaths) and DU (Unreported Deaths). This model is implemented using the R package SEIRfansy [26].

##### Formulation

**Fig. 4:**
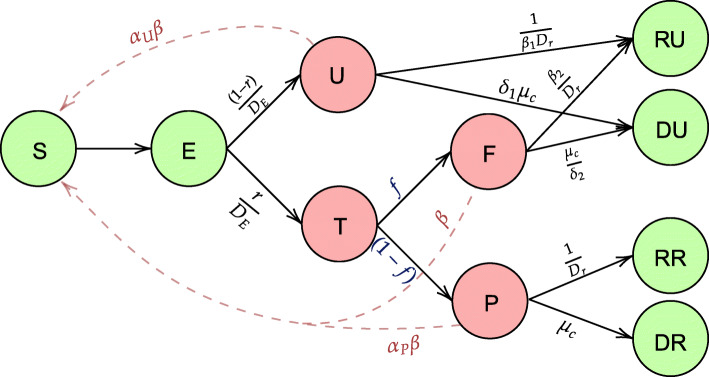
Schematic diagram for the SEIR-fansy model with imperfect testing and misclassification. The model has ten compartments: S (Susceptible), E (Exposed), T (Tested), U (Untested), P (Tested positive), F (Tested False Negative), RR (Reported Recovered), RU (Unreported Recovered), DR (Reported Deaths) and DU (Unreported Deaths). Reproduced from Bhaduri, Kundu et al., 2020 [18]

Like most compartmental models, this model assumes exponential times for the duration of an individual staying in a compartment. For simplicity, we approximate this continuous-time process by a discrete-time modeling process. The main parameters of this model are *β* (rate of transmission of infection by false negative individuals), *α*_*p*_ (scaling factor that measures the rate of spread of infection by patients who test positive for COVID-19 relative to infected patients who return false negative test results), *α*_*u*_ (scaling factor for the rate of spread of infection by untested individuals), *D*_*e*_ (incubation period in days), *D*_*r*_ (mean days till recovery for positive individuals), *D*_*t*_ (mean number of days for the test result to come after a person is being tested), *μ*_*c*_ (death rate due to COVID-19 which is the inverse of the average number of days for death due to COVID-19 starting from the onset of disease multiplied by the probability death of an infected individual due to COVID), *λ* and *μ* (natural birth and death rates respectively, assumed to be equal for the sake of simplicity), *r* (probability of being tested for infectious individuals), *f* (false negative probability of RT-PCR test), ${\beta}_1\  and\ {\beta}_2^{-1}$ (scaling factors for rate of recovery for undetected and false negative individuals respectively), ${\delta}_1\mathrm{and}\ {\delta}_2^{-1}$ (scaling factors for death rate for undetected and false negative individuals respectively). The number of individuals at the time point *t* in each compartment is governed by the system of differential equations given by Eqs. (8a) – (8i). To simplify this model, we assume that testing is instantaneous. In other words, we assume there is no time difference from the onset of the disease after the incubation period to getting test results. This is a reasonable assumption to make as the time for testing is about 1–2 days which is much less than the mean duration of stay for the other compartments. Further, once a person shows symptoms for COVID-19 like diseases, they are sent to get tested almost immediately. Figure 4 provides a schematic overview of the model.

The following differential equations summarize the transmission dynamics being modeled.

8a $$ \frac{\partial S}{\partial t}=-\beta \frac{S(t)}{N}\left({\alpha}_PP(t)+{\alpha}_UU(t)+F(t)\right)+\lambda N-\mu S(t), $$

8b $$ \frac{\partial E}{\partial t}=\beta \frac{\mathrm{S}(t)}{N}\left({\alpha}_PP(t)+{\alpha}_UU(t)+F(t)\right)-\frac{E(t)}{D_e}-\mu E(t), $$

8c $$ \frac{\partial U}{\partial t}=\left(1-r\right)\frac{E(t)}{D_e}-\frac{U(t)}{\beta_1{D}_r}-{\delta}_1{\mu}_cU(t)-\mu U(t), $$

8d $$ \frac{\partial P}{\partial t}=\left(1-f\right)r\frac{E(t)}{D_e}-\frac{P(t)}{D_r}-{\mu}_cP(t)-\mu P(t), $$

8e $$ \frac{\partial F}{\partial t}= fr\frac{E(t)}{D_e}-\frac{\beta_2F(t)}{D_r}-\frac{\mu_cF(t)}{\delta_2}-\mu F(t), $$

8f $$ \frac{\partial RU}{\partial t}=\frac{U(t)}{\beta_1{D}_r}+\frac{\beta_2F(t)}{D_{\mathrm{r}}}-\mu RU(t), $$

8g $$ \frac{\partial RR}{\partial t}=\frac{P(t)}{D_r}-\mu RR(t), $$

8h $$ \frac{\partial DU}{\partial t}={\delta}_1{\mu}_cU(t)+\frac{\mu_cF(t)}{\delta_2}, $$

8i $$ \frac{\partial DR}{\partial t}={\mu}_cP(t). $$

Using the Next Generation Matrix Method [41], we calculate the basic reproduction number

9 $$ {R}_0=\frac{\beta {S}_0}{\mu {D}_e+1}\left(\frac{\alpha_U\left(1-r\right)}{\frac{1}{\beta_1{D}_r}+{\delta}_1{\mu}_c+\mu }+\frac{\alpha_Pr\left(1-f\right)}{\frac{1}{D_r}+{\mu}_c+\mu }+\frac{rf}{\frac{\beta_2}{D_r}+\frac{\mu_c}{\delta_2}+\mu}\right), $$

where *S*_0_ = λ/μ = 1 since we assume that natural birth and death rates are equal within this short period of time. Supplementary Table S1 describes the parameters in greater detail.

##### Likelihood assumptions and estimation

Parameters are estimated using Bayesian estimation techniques and MCMC methods (namely, Metropolis-Hastings method [42] with Gaussian proposal distribution). First, we approximated the above set of differential equations by a discrete time approximation using daily differences. After we start with an initial value for each of the compartments on the day 1, using the discrete time recurrence relations we obtain the counts for each of the compartments at the next days. To proceed with the MCMC-based estimation, we specify the likelihood explicitly. We assume (conditional on the parameters) the number of new confirmed cases on day *t* depend only on the number of exposed individuals on the previous day. Specifically, we use multinomial modeling to incorporate the data on recovered and deceased cases as well. The joint conditional distribution is

$$ P\left[{P}_{new}(t),{RR}_{new}(t),{DR}_{new}(t)\right|E\left(t-1\right),P\left(t-1\right)\left]=P\left[{P}_{new}(t)\right|E\left(t-1\right),P\left(t-1\right)\right].P\left[\ {RR}_{new}(t),{DR}_{new}(t)\right|E\left(t-1\right),P\left(t-1\right)\left]=P\left[{P}_{new}(t)\right|E\left(t-1\right)\right].P\left[\ {RR}_{new}(t),{DR}_{new}(t)\right|P\left(t-1\right)\Big]. $$

A multinomial distribution-like structure is then defined

10a $$ {P}_{new}(t)\mid E\left(t-1\right)\sim Bin\left(E\left(t-1\right),r\left(1-f\right)/{D}_e\right) $$

10b $$ {RR}_{new}(t),{DR}_{new}(t)\mid P\left(t-1\right)\sim Mult\left(P\left(t-1\right),\left({D}_r^{-1},{\mu}_c,1-{D}_r^{-1}-{\mu}_c\right)\right) $$

*Note:* the expected values of *E*(*t* − 1) and *P*(*t* − 1) are obtained by solving the discrete time differential equations specified by Eqs. (8a) – (8i).

##### Prior assumptions and MCMC

For the parameter *r*, we assume a *U*(0, 1) prior, while for *β*, we assume an improper non-informative flat prior with the set of positive real numbers as support. After specifying the likelihood and the prior distributions of the parameters, we draw samples from the posterior distribution of the parameters using the Metropolis-Hastings algorithm with a Gaussian proposal distribution. We run the algorithm for 200,000 iterations with a burn-in period of 100,000. Finally, the mean of the parameters in each of the iterations are obtained as the final estimates of *β* and *r* for the different time periods. As in the case of the SAPHIRE model, we again break the training period into ten sequential blocks: pre-lockdown (March 15–24), lockdown phases 1, 2, 3, and 4 (March 25 – April 14, April 15 – May 3, May 4–17, and May 18–31 respectively) followed by unlock phases 1, 2, 3, 4 and 5 (June 1–30, July 1–31, August 1–31, September 1–30 and October 1–15 respectively).

#### Imperial College London model (ICM)

##### Overview

**Fig. 5:**
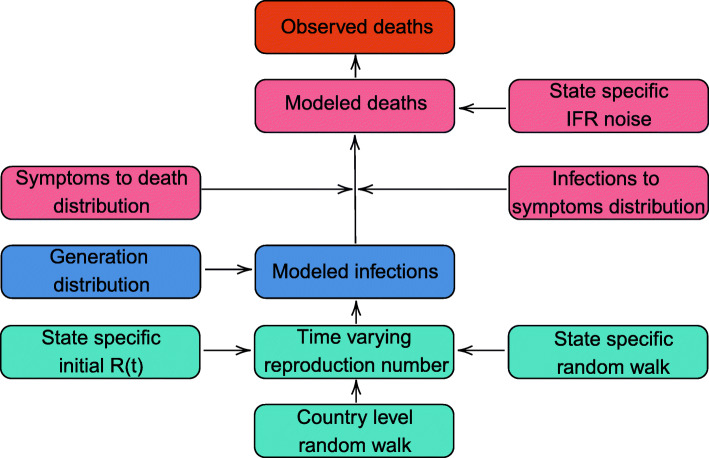
Schematic overview of ICM

We examine a Bayesian semi-mechanistic model for estimating the transmission intensity of SARS-CoV-2 [7]. The model defines a renewal equation using the time-varying reproduction number *R*_*t*_ to generate new infections. As a lot of cases in SARS-CoV-2 are asymptomatic and reported case data is unreliable especially in early part of the epidemic in India, the model relies on observed deaths data and calculates backwards to infer the true number of infections. The latent daily infections are modeled as the product of *R*_*t*_ with a discrete convolution of the previous infections, weighted using an infection-to-transmission distribution specific to SARS-CoV-2. We implement this Bayesian semi-mechanistic model in the context of COVID-19 data arising from India in order to estimate the reproduction number over time, along with plausible upper and lower bounds (95% Bayesian credible intervals (CrI)) of the daily infections and the daily number of infectious people. We parametrize *R*_*t*_ with a fixed effect and a random effect for each week over the course of the epidemic for each state. The fixed effect accounts for the variations in *R*_*t*_ across India as a whole whereas the random effect allows for variations among different states. The weekly effects are encoded as a random walk, where at each successive step the random effect has an equal chance of moving upwards or downwards from its current value. The model is implemented using epidemia [43], a general purpose R package for semi-mechanistic Bayesian modelling of epidemics. Figure 5 represents a schematic overview of the model.

##### Formulation

The true number of infected individuals, *i*, is modelled using a discrete renewal process. We specify a generation distribution [44] *g* with density *g*(τ) as *g* ∼ Gamma(6.5,0.62). Given the generation distribution, the number of infections *i*_*t*, *m*_ on a given day *t*, and state *m* is given by the discrete. Convolution function:

11a $$ {i}_{t,m}={S}_{t,m}{R}_{t,m}\sum \limits_{\uptau =0}^{t-1}{i}_{\uptau, m}{g}_{t-\uptau}, $$

11b $$ {S}_{t,m}=1-\frac{\sum_{j=0}^{t-1}{i}_{j,m}}{N_m}, $$

where the generation distribution is discretized by ${g}_s={\int}_{s-0.5}^{s+0.5}g\left(\uptau \right)d$ for *s* = 2, 3, …,and ${g}_1={\int}_0^{1.5}g\left(\uptau \right)d\uptau$. The population of state *m* is denoted by *N*_*m*_. We include the adjustment factor *S*_*t*, *m*_ to account for the number of susceptible individuals left in the population.

We define daily deaths, *D*_*t*, *m*_, for days *t* ∈ {1, …, *n*} and states *m* ∈ {1, …, *M*}. These daily deaths are modelled using a positive real-valued function *d*_*t*, *m*_ = *E*[*D*_*t*, *m*_] that represents the expected number of deaths attributed to COVID-19. The daily deaths *D*_*t*, *m*_ are assumed to follow a negative binomial distribution with mean *d*_*t*, *m*_ and variance ${d}_{t,m}+{d}_{t,m}^2/{\uppsi}_1$, where ψ_1_ follows a positive half normal distribution, i.e.,

12a $$ {D}_{t,m}\sim \mathrm{NB}\ \left({d}_{t,m},{d}_{t,m}+{d}_{t,m}^2/{\uppsi}_1\right),\kern1em t=1,\dots, n, $$

12b $$ {\uppsi}_1\sim {N}^{+}\left(0,5\right). $$

We link our observed deaths mechanistically to transmission [7]. We use a previously estimated COVID-19 infection fatality ratio (IFR, probability of death given infection) of 0.1% [45, 46] together with a distribution of times from infection to death π. To incorporate the uncertainty inherent in this estimate we modify the ifr for every state to have additional noise around the mean, denoted by $\mathrm{if}{\mathrm{r}}_{\mathrm{m}}^{\ast }$. Specifically, we assume.

13 $$ \mathrm{if}{\mathrm{r}}_{\mathrm{m}}^{\ast}\sim \mathrm{if}\mathrm{r}\cdotp N\left(1,0.1\right), $$

where $\mathrm{if}{\mathrm{r}}_{\mathrm{m}}^{\ast }$ represents the noise-added analog of ifr. Using estimated epidemiological information from previous studies, we assume the distribution of times from infection to death π (infection-to-death) to be the convolution of an infection-to-onset distribution (π^′^) [47] and an onset-to-death distribution [32].

14 $$ \uppi \sim \mathrm{Gamma}\left(5.1,0.86\right)+\mathrm{Gamma}\left(17.8,0.45\right). $$

The expected number of deaths *d*_*t*, *m*_, on a given day *t*, for state *m* is given by the following discrete sum

15 $$ {d}_{t,m}=\mathrm{if}{\mathrm{r}}_{\mathrm{m}}^{\ast}\sum \limits_{\uptau =0}^{t-1}{i}_{\uptau, m}{\uppi}_{t-\uptau}, $$

where *i*_*τ*, *m*_ is the number of new infections on day *τ* in state *m* and where, similar to the generation distribution, *π* is discretized via ${\uppi}_s={\int}_{s-0.5}^{s+0.5}\uppi \left(\uptau \right)d\uptau$ for *s* = 2, 3, …, and ${\uppi}_1={\int}_0^{1.5}\uppi \left(\uptau \right)\mathrm{d}\uptau$, where π(τ) is the density of π.

We parametrize *R*_*t*, *m*_ with a random effect for each week of the epidemic as follows

16 $$ {R}_{t,m}={R}_0\cdotp f\left(-{\upepsilon}_{w\left(t,m\right)}-{\upepsilon}_{m,w\left(t,m\right)}^{state}\right), $$

where *f*(*x*) = 2 *exp* (*x*)/(1 +  *exp* (*x*)) is twice the inverse logit function, and ϵ_*w*(*t*)_ and ${\epsilon}_{m,w\left(t,m\right)}^{state}$follow a weekly random walk process, that captures variation between *R*_*t*, *m*_ in each subsequent week. *ϵ*_*w*(*t*)_ is a fixed effect estimated across all the states and ${\epsilon}_{m,w\left(t,m\right)}^{state}$ is the random effect specific to each state in India. The prior distribution for *R*_0_ [30] was chosen to be

17 $$ {R}_0\sim N\left(\mathrm{3.28,0.5}\right). $$

We assume that seeding of new infections begins 30 days before the day after a state has cumulatively observed 10 deaths. From this date, we seed our model with 6 sequential days of an equal number of infections: *i*_1_ = … = *i*_6_ ∼ Exponential(τ^−1^), where τ ∼ Exponential(0.03). These seed infections are inferred in our Bayesian posterior distribution. Fitting was done with the R package epidemia [43] which uses STAN [48], a probabilistic programming language, using an adaptive Hamiltonian Monte Carlo (HMC) sampler.

### Comparing models and evaluating performance

Having established differences in the formulation of the different models, we compare their respective projections and inferences. In order to do so, we use the same data sources [49, 50] for all five models. Well-defined time points are used to denote training (March 15 to October 15) and test (October 16 to December 31) periods.

Using the parameter values specified above along with data from the training period as inputs, we compare the projections of the five models with observed data from the test period. In order to do so, we use the symmetric mean absolute prediction error (SMAPE) and mean squared relative prediction error (MSRPE) metrics as measures of accuracy. Given observed time-varying data ${\left\{{O}_t\right\}}_{t=1}^T$ and an analogous time-series dataset of projections ${\left\{{P}_t\right\}}_{t=1}^T$, the SMAPE metric is defined as

18 $$ SMAPE(T)=\frac{100}{T}\cdotp \sum \limits_{t=1}^{t=T}\frac{\left|{P}_t-{O}_t\right|}{\left(\left|{P}_t\right|+\left|{O}_t\right|\right)/2}, $$

where |*x*| denotes the absolute value of *x*. The metric MSRPE is defined as

19 $$ MSRPE(T)={\left[{T}^{-1}\sum \limits_{t=1}^T{\left(1-\frac{P_t}{O_t}\right)}^2\right]}^{1/2}. $$

It can be seen that 0 ≤ *SMAPE* ≤ 100, with smaller values of both MSRPE and SMAPE indicating a more accurate fit. For active reported cases (cases that are active on a given day which is the difference of cumulative reported cases and cumulative reported counts of recoveries and deaths), we compute and compare the metrics defined above for projections from eSIR and SEIR-fansy models as no other model returns relevant projections. For cumulative reported cases we obtain projections from all models apart from ICM (which yields total, i.e., sum of reported and unreported, cumulative cases). For cumulative reported deaths we compare projections from eSIR, SEIR-fansy and ICM, since the baseline and SAPHIRE models do not yield relevant projections. Supplementary Table S2 gives an overview of output from each of the models we consider and Table 2 reports the values of accuracy metrics described above.

**Table 2:** Comparison of estimated time-varying *R*_*t*_ and prediction accuracy of the models under consideration

	Model				
	Baseline^**a**^	eSIR	SAPHIRE^**b**^	SEIR-***fansy***	ICM^**c**^
Estimated mean reproduction number ***R*** [95% CrI]					
**Lockdown 1.0** ***(March 25 – April 14)***	–	2.12 [1.44, 2.16]	2.54 [2.41, 2.74]	5.03 [5.01, 5.04]	1.77 [1.58, 1.96]
**Lockdown 2.0** ***(April 15 – May 3)***		1.48 [1.00, 1.51]	1.60 [1.36, 2.17]	1.90 [1.89, 1.91]	1.22 [1.18, 1.27]
**Lockdown 3.0** ***(May 4 – May 17)***		0.87 [0.59, 0.89]	1.69 [1.46, 1.97]	2.71 [2.67, 2.73]	1.33 [1.28, 1.38]
**Lockdown 4.0** ***(May 18 – May 31)***		0.89 [0.61, 0.91]	1.54 [1.29, 2.00]	2.33 [2.30, 2.36]	1.41 [1.35, 1.47]
**Unlock 1.0** ***(June 1 – June 30)***		0.85 [0.58, 0.87]	1.27 [1.19, 1.32]	1.74 [1.73, 1.75]	1.05 [0.99, 1.10]
**Unlock 2.0** ***(July 1 – July 31)***		0.77 [0.52, 0.78]	1.31 [1.22, 1.36]	1.80 [1.79, 1.81]	1.11 [1.08, 1.14]
**Unlock 3.0** ***(August 1 – August 31)***		0.79 [0.54, 0.81]	1.16 [1.06, 1.31]	1.25 [1.24, 1.26]	1.05 [1.04, 1.07]
**Unlock 4.0** ***(September 1 – September 30)***		0.69 [0.47, 0.7]	1.12 [0.98, 1.49]	1.06 [1.05, 1.07]	0.89 [0.86, 0.91]
**Unlock 5.0** ***(October 1 – October 15)***		0.67 [0.45, 0.68]	1.09 [0.91, 1.69]	0.86 [0.85, 0.87]	0.83 [0.82, 0.84]
Prediction accuracy using %-SMAPE (MSRPE)^d^					
**Active reported cases**	–	37.955 (2.283)	–	35.141 (1.114)	–
**Cumulative reported cases**	6.889 (0.173)	6.593 (0.198)	2.250 (0.056)	2.285 (0.048)	
**Cumulative reported deaths**	–	8.943 (0.253)	–	4.737 (0.115)	0.771 (0.020)

^a^The baseline model does not return estimates of time-varying *R*(*t*) or projections of active reported cases or cumulative reported deaths

^b^The SAPHIRE model does not return projections of active reported cases or cumulative reported deaths

^c^The ICM model does not return projections of active or cumulative reported cases

^d^We compare model projections with observed reported data from October 16 till December 31, 2020

Further, we compare (when possible) the estimated time-varying reproduction number *R*(*t*) over the different lockdown and unlock stages in India. Specifically, for each lockdown stage, we report the median *R*(*t*) value along with the associated 95% credible interval (CrI). The values are presented in Table 2.

Since we are interested in comparing relative performances of the models (specifically, their projections), we define another metric – the relative mean squared prediction error (Rel-MSPE). Given time series data on observed cumulative cases (or deaths) ${\left\{{O}_t\right\}}_{t=1}^T$, projections from a model A ${\left\{{P}_t^A\right\}}_{t=1}^T$, and projections from some other model B, ${\left\{{P}_t^B\right\}}_{t=1}^T$, the Rel-MSPE of model B with respect to model A is defined as

20 $$ Rel- MSPE\left(B:A\right)={\left[\sum \limits_{t=1}^T{\left(\frac{O_t-{P}_t^A}{O_t-{P}_t^B}\right)}^2\right]}^{1/2} $$

Higher values of Rel-MSPE(B:A) indicate better performance of model B over model A. Since the baseline model yields projections of cumulative reported cases, we compute Rel-MSPE for the other models with respect to the baseline model for reported cumulative cases. Projections from ICM represent total (i.e., sum of reported and unreported) cumulative cases and are left out of this comparison of reported counts. For cumulative reported deaths, we compute Rel-MSPE of the SEIR-fansy and ICM models relative to the eSIR model. In addition to comparing the accuracy of fits that arise from the different models, we also investigate if projections from the different models are correlated with observed data. We use the standard Pearson’s correlation coefficient and Lin’s concordance correlation coefficient [51] as summary measures to study said correlation. Higher values of these correlation metrics indicate better concordance of model projections and the observed data from the test period. Rel-MSPE and correlation metrics are presented in Table 3. Since we have projections for total (sum of reported and unreported cases) for active cases from SEIR-fansy, for cumulative cases from SAPHIRE, SEIR-fansy and ICM, and for cumulative deaths from SEIR-fansy, we present the projected totals along with 95% credible intervals and associated underreporting factors on three specific dates – October 31, November 30 and December 31 in Table 4. The table also includes projected cumulative reported counts (which are available from all models under investigation apart from ICM) with 95% credible intervals for the three dates mentioned above.

**Table 3:** Comparison of relative performance and correlation with observed data of projections of the models under consideration from October 16 till December 31, 2020

Observed data (confirmed)	Metric	Model				
		Baseline	eSIR	SAPHIRE	SEIR-***fansy***	ICM^**e**^
Cumulative cases	**Rel-MSPE**^**a**^	1	1.724	3.013	3.270	–
	**Pearson’s correlation coefficient**^**b**^	0.996	0.969	0.984	0.999	
	**Lin’s concordance coefficient**^**b**^	0.507	0.476	0.738	0.891	
Cumulative deaths	**Rel-MSPE**^**c**^	–	1	–	6.962	3.64
	**Pearson’s correlation coefficient**^**d**^		1		1	0.996
	**Lin’s concordance coefficient**^**d**^		0.339		0.616	0.956

^a^For cumulative reported cases, Rel-MSPE is defined relative to projections from the baseline model

^b^For cumulative reported cases, the correlation coefficients of the projections are compared with respect to observed data

^c^For cumulative reported deaths, Rel-MSPE is defined relative to projections from the eSIR model

^d^For cumulative reported deaths, the correlation coefficients of the projections are compared with respect to observed data

^e^The ICM model returns total (reported + unreported) cumulative case counts, so we leave it out of our comparisons

**Table 4:** Projected counts of reported cumulative cases and total (sum of reported and unreported) counts of cases and deaths (cumulative) from the models under comparison

Counts	Model	October 31, 2020	November 30, 2020	December 31, 2020
Projected cumulative reported counts (95% CrI) for specific dates in test period^c^				
Cumulative cases (in millions)	Observed	8.18	9.46	10.29
	Baseline	8.71 (8.63–8.80)	11.12 (10.83–11.43)	13.34 (12.81–13.93)
	eSIR	8.35 (7.19–9.60)	10.91 (8.38–13.93)	14.85 (9.88–21.81)
	SAPHIRE	8.17 (7.90–8.52)	8.93 (8.17–9.67)	9.26 (8.19–10.35)
	SEIR-fansy	8.51 (8.18–8.85)	9.91 (9.54–10.30)	10.97 (10.57–11.4)
Projected total counts^a^ (95% CrI) [under-reporting factor^b^] for specific dates in test period^c^				
Active cases (in millions)	Observed	0.57	0.44	0.26
	SEIR-fansy	5.32 (5.12–5.52) [9.3]	3.99 (3.85–4.14) [9.13]	2.96 (2.85–3.06) [11.53]
Cumulative cases (in millions)	Observed	8.18	9.46	10.29
	SAPHIRE^d^	578.21 (46.41–1134.20) [70.7]	612.79 (52.253–1161.26) [64.8]	622.32 (55.79–1163.17) [60.5]
	SEIR-fansy	59.32 (56.8–61.72) [7.25]	68.71 (65.95–71.47) [7.26]	75.89 (72.89–78.86) [7.38]
	ICM^d^	37.17 (24.78–58.68) [4.54]	39.54 (25.63–63.12) [4.18]	41.38 (26.02–67.88) [4.02]
Cumulative deaths (thousands)	Observed	121.56	137.07	148.43
	SEIR-fansy	361.52 (347.23–375.85) [2.97]	442.25 (425.05–459.64) [3.23]	504.76 (485.50–524.07) [3.4]

^a^Projected total count includes both reported as well as unreported values

^b^Defined as projected total/observed reported counts, where total is the sum of reported and unreported cases

^c^The test period extends from October 16 till December 31, 2020. We examine projections of cumulative cases and counts on three specific dates within that period, namely, October 31, November 30 and December 31, 2020

^d^The SAPHIRE model does not yield projections of active cases or cumulative deaths while the ICM model does not yield projections of cumulative reported cases, total active cases or total cumulative deaths

### Data source

The data on confirmed cases, recovered cases and deaths for India and the 20 states of interest are taken from COVID-19 India [49] and the JHU CSSE COVID-19 GitHub repository [50]. In addition to this and other similar articles concerning the spread of this disease in India, we have created an interactive dashboard [52] summarizing COVID-19 data and forecasts for India and its states (generated with the eSIR model discussed in this paper). While the models are trained using data from March 15 to October 15, 2020, their performances are compared by examining their respective projections from October 16 to December 31, 2020.

## Results

### Estimation of the reproduction number

From Table 2, we compare the mean of the time-varying effective reproduction number *R*(*t*) over the four phases of lockdown and subsequent unlock phased in India. The eSIR model returns a mean value of 2.08 (95% credible interval: 1.41–2.12) over the entire training period. Factoring in different levels of government interventions which modified transmission dynamics during lockdown, we get period specific estimates ranging from 2.12 (1.44–2.16) in lockdown phase 1, which drops to 1.48 (1.00–1.51) in lockdown phase 2 and then reports a steady decline over the subsequent lockdown and unlock phases. The mean values returned by the SAPHIRE model varied from 2.54 (2.41–2.74) during phase 1 of the lockdown, 1.60 (1.36–2.17) for phase 2, 1.69 (1.46–1.97) for phase 3 and 1.54 (1.29–2.00) for the fourth and final lockdown phase. The estimated values for subsequent unlock phases are quite close to each other, starting from 1.27 (1.19–1.32) in unlock phase 1 and dropping to 1.09 (0.91–1.69) in the fifth unlock phase. The SEIR-fansy notes that the mean *R*(*t*) drops from 5.03 (5.01–5.04) during the first phase of lockdown, to 1.90 (1.89–1.91) during the second lockdown phase, before rising again to 2.33 (2.30–2.36) during lockdown phase 4. The estimated mean drops steadily from 1.80 (1.79–1.81) during unlock phase 2 to 0.86 (0.85–0.87) during unlock phase 5. The ICM-based mean values fluctuate, from 1.77 (1.58–1.96) during the first lockdown phase, followed by 1.22 (1.18–1.27), then dropping to 1.33 (1.28–1.38) and finally rising to 1.41 again (1.35–1.47) for the fourth phase of lockdown. Estimates from ICM during unlock phases behave like those from the SEIR-fansy model – in unlock phase 2 the estimated mean is 1.11 (1.08–1.14) and in unlock phase 5, the mean is 0.83 (0.82–0.84). In terms of agreement of reported values, SAPHIRE, SEIR-fansy and ICM report the highest mean R for phase one of the lockdown. Values reported by SAPHIRE, SEIR-fansy and ICM report a drop in intermediate lockdown phases, followed by a rise. Values during unlock period increase from phase 1 to phase 2, followed by a steady decline. SAPHIRE, SEIR-fansy and ICM report the lowest value of R for unlock phase 5.

### Estimation of reported case counts

**Fig. 6:**
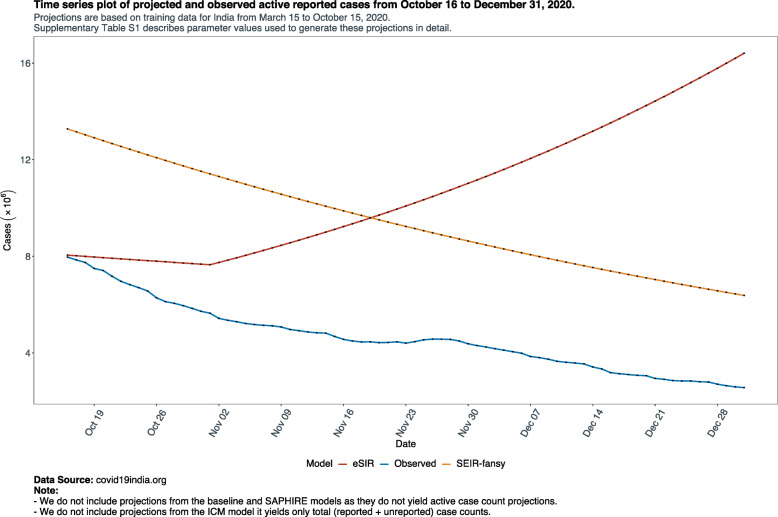
Comparison of projected and observed reported active cases from October 16 to December 31 for India, using training data from March 15 to October 15, 2020

**Fig. 7:**
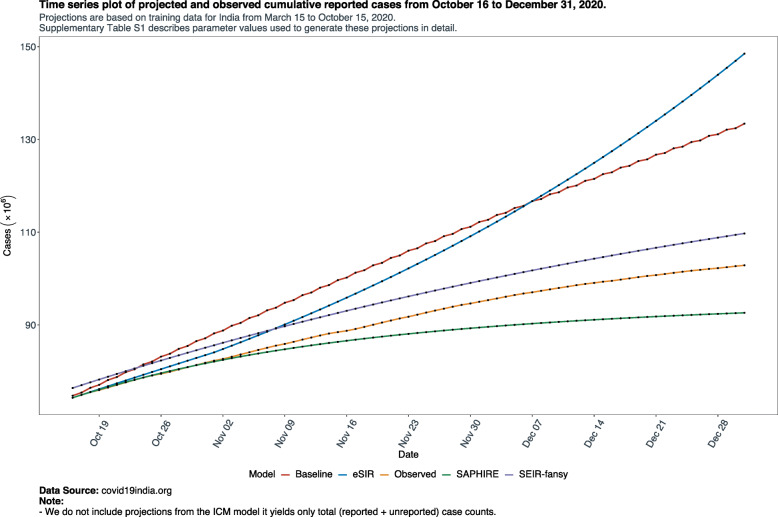
Comparison of projected and observed reported cumulative cases from October 16 to December 31 for India, using training data from March 15 to October 15, 2020

**Fig. 8:**
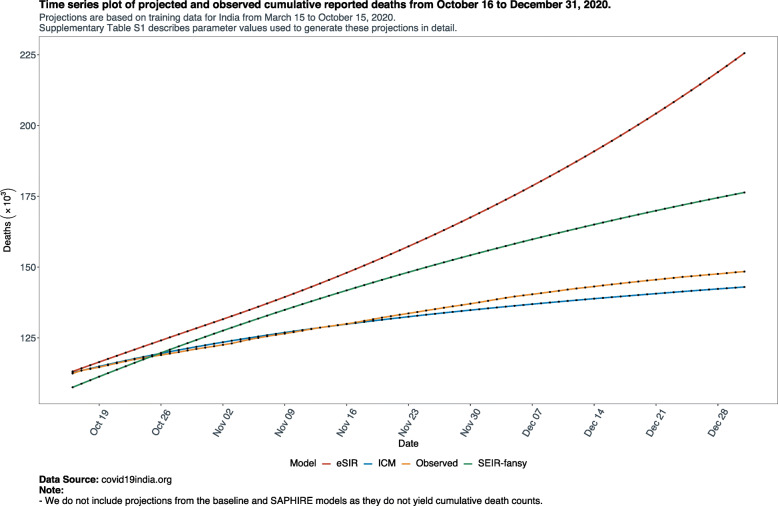
Comparison of projected and observed reported cumulative deaths from October 16 to December 31 for India, using training data from March 15 to October 15, 2020

**Fig. 9:**
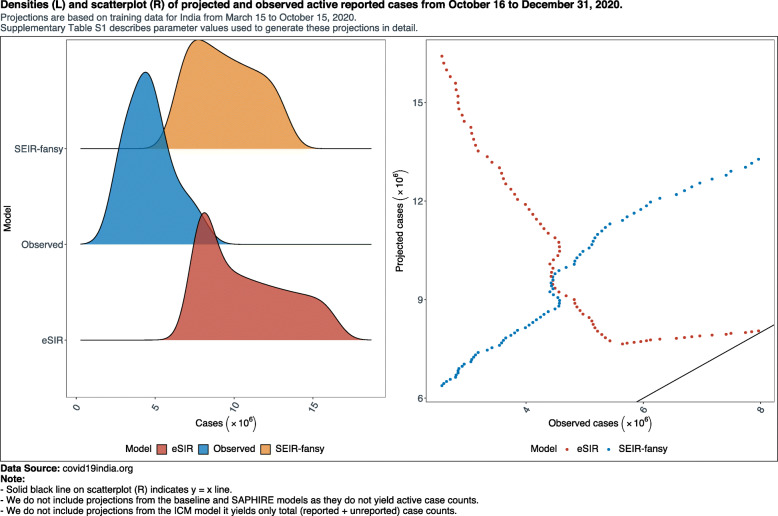
Scatter plot and marginal densities of projected and observed reported active cases from October 16 to December 31 for India, using training data from March 15 to October 15, 2020

From Figs. 6, 7, 8 and 9, we note that the eSIR model overestimates the count of active cases – a behavior which gets worse with time. While the observed counts decrease steadily in the test period, the eSIR model fails to capture this behaviour and returns projections which rise over time. In comparison, the SEIR-fansy model is able to replicate the decreasing behaviour but yields projections which are higher than observed counts. In terms of prediction accuracy, the SEIR-fansy model has an SMAPE value of 35.14% and an MSRPE value of 1.11. For eSIR model, those values are at 37.96% (SMAPE) and 2.28 (MSRPE).

**Fig. 10:**
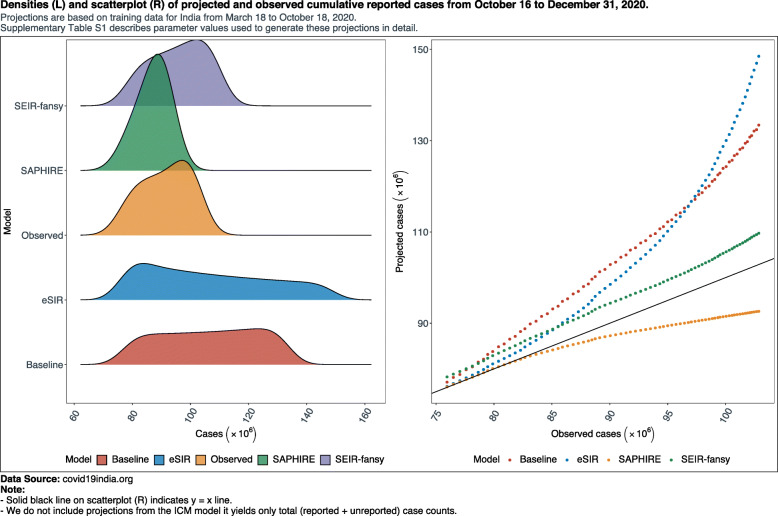
Scatter plot and marginal densities of projected and observed cumulative cases from October 16 to December 31 for India, using training data from March 15 to October 15, 2020

From Figs. 7, 8, 9 and 10 we note that while the SAPHIRE model underestimates the count of cumulative cases, the baseline, eSIR and SEIR-fansy models overestimate the count. Table 2 reveals that SAPHIRE performs the best in terms of SMAPE metric with a value of 2.25%, followed closely by SEIR-fansy (2.29%). The eSIR and baseline models perform poorly in comparison, yielding 6.59 and 6.89% respectively. The SEIR-fansy model performs best in terms of MSRPE with a value of 0.05, followed closely by SAPHIRE (0.06)*.* Table 3 further reveals a similar relative performance through Rel-MSPE values (all Rel-MSPE figures reported here are relative to the baseline model). The SEIR-fansy model performs the best with Rel-MSPE value of 3.27, followed by SAPHIRE (3.01), and finally, the eSIR model (1.72). All four sets of projections are highly correlated with the observed time series – with all model projections having a Pearson’s correlation coefficient of nearly 1 with the observed data. Lin’s concordance coefficient yields an ordering (from worst to best) of the eSIR model (0.48), followed by the baseline model (0.51), the SAPHIRE model (0.74) and finally, the SEIR-fansy model (0.89).

### Estimation of reported death counts

**Fig. 11:**
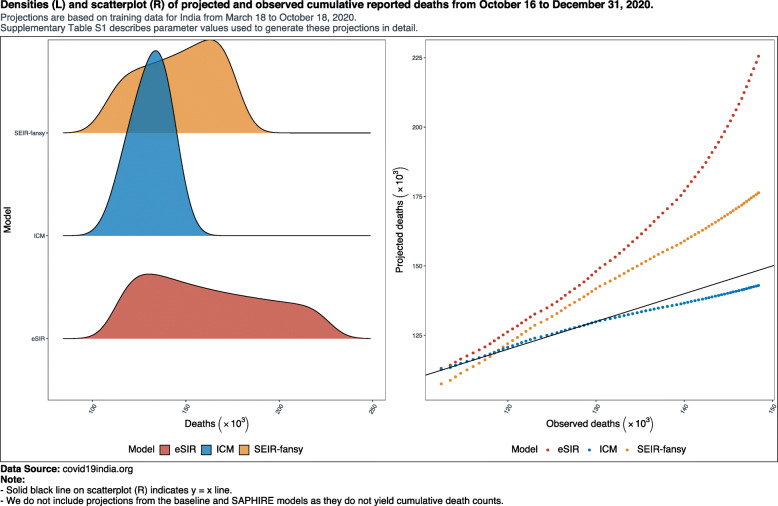
Scatter plot and marginal densities of projected and observed cumulative death from October 16 to December 31 for India, using training data from March 15 to October 15, 2020

From Figs. 8, 9, 10 and 11, we note that the eSIR and SEIR-fansy models almost always overestimate, whereas the ICM model slightly underestimates the confirmed cumulative death counts. From Table 2 and Table 3, the SMAPE and MSRPE values, along with comparison of projections with observed data reveal that the ICM model is most accurate (SMAPE: 0.77%, MSRPE: 0.020), followed by SEIR-fansy (SMAPE: 4.74%, MSRPE: 0.12) followed by the eSIR model (SMAPE: 8.94%, MSRPE: 0.25). Relative to the eSIR model, the Rel-MSPE values of the models reveal that the SEIR-fansy model performs better (Rel-MSPE: 6.96), followed by ICM (Rel-MSPE: 3.64). Judging by values of Pearson’s correlation coefficient, all three sets of projections are highly correlated with the observed data. Lin’s concordance coefficient yields an ordering (from best to worst) of ICM (0.96), followed by SEIR-fansy (0.62) and finally eSIR (0.34).

### Estimation of unreported case and death counts

From Table 4, we note that the SEIR-fansy model yields underreporting factors of about 10 for active cases on October 31, November 30 and December 31. Further, we observe that the SAPHIRE model projects the maximum count of total cumulative cases on the above three dates, followed by the SEIR-fansy and then ICM. SAPHIRE returns under-reporting factors of the order of approximately 65, while SEIR-fansy and ICM return under-reporting factors which are approximately 7 and 4 respectively. For cumulative deaths, SEIR-fansy estimates underreporting factors approximately equal 3.

### Uncertainty quantification of estimates and predictions

**Fig. 12:**
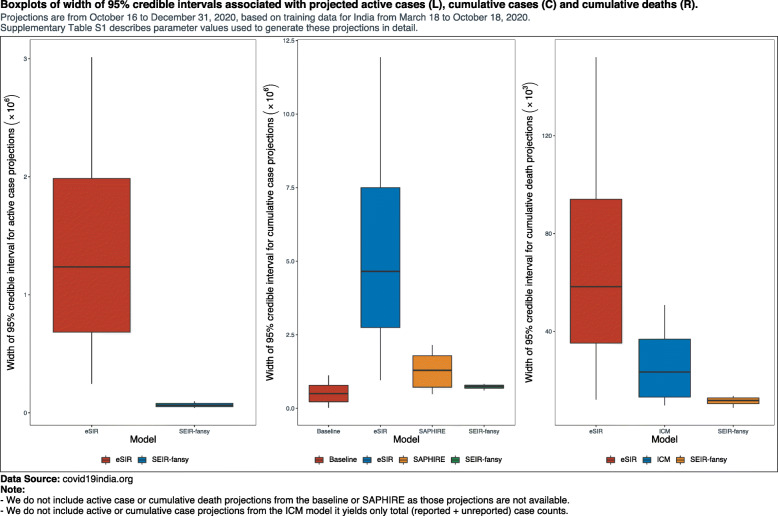
Boxplots showing width of 95% credible interval associated with projected active cases, cumulative cases and cumulative deaths from October 16 to December 31 for India, using training data from March 15 to October 15, 2020

From Fig. 12 we observe that the width of 95% credible intervals associated with projections from each of the models vary significantly. While the eSIR model consistently returns the widest intervals, SEIR-fansy has the narrowest intervals. In case of cumulative counts, the ordering (best to worst) starts with SEIR-fansy, followed by the baseline, followed by SAPHIRE and finally the eSIR model. For cumulative deaths, the ordering (best to worst) starts with SEIR-fansy, followed by ICM and finally eSIR. From Table 4, we compare projections of reported cumulative cases for each model (apart from ICM which returns projections of cumulative total cases and not cumulative reported cases) and their associated prediction intervals on October 31, November 30 and December 31, 2020. On October 31, we observe 8.18 million cumulative reported cases, while the projections (in millions) from the baseline model are 8.71 (95% credible interval: 8.63–8.80), while eSIR yields 8.35 (7.19–9.60), SAPHIRE returns 8.17 (7.90–8.52) and SEIR-fansy projects 8.51 (8.18–8.85) million cases. We do not present our projections for November 30 and December 31, 2020 here in the interest of conciseness.

## Sensitivity analyses and performance in other countries

Sensitivity analyses for some of the discussed models have been carried out in several other publications. In the interest of conciseness, we refer to said publications and comment on what parameters are central to estimation and generating projections for the models examined here. We also include information on how these models have performed in the context of data from other countries.

### eSIR

Evaluation of the model results in terms of their sensitivity to initial parameter choices and under-reporting and clustering issues within the data have been discussed in the context of India in prior literature [53]. The range of scenarios considered earlier include 10-fold underreporting of cases, clustering of cases in metropolitan areas, and prior mean of *R*_0_ ranging from 2 to 4 (See Supplementary Table S3). Even though the posterior estimates and predictions changed in scale to some extent across these scenarios, they did not significantly change the broad conclusions. It is undeniable that the exact predicted case counts are sensitive to the choice of priors, but with new data coming in over a longer time frame, as seen in the results from this work, the model is capable of washing out the prior effects in the posterior outcomes.

The eSIR model has been successfully implemented and utilized in the context of COVID-19 across different geographical locations, including China [24, 25, 54], Poland [55], Italy [24], Bangladesh and Pakistan [56]. These countries cover a broad range in terms of socio-economic status, health infrastructure and pandemic management strategies. In each of these cases the eSIR model was seen to be successfully capturing the patterns of growth of the pandemic via estimated parameters, as well as efficiently forecasting future case counts via predictive modeling.

### SAPHIRE

We conducted the sensitivity analysis (results not shown) by changing the initial parameters as 20% lower or higher than the specified values in the SAPHIRE model. The estimated *R* and ascertainment rates were robust to misspecification of the duration from the onset of symptoms to isolation and of the relative transmissibility of unreported versus reported cases. *R* estimates were positively correlated with the specified latent and infectious periods, and the estimated ascertainment rates were positively correlated with the specified ascertainment rate in the initial state. This finding is consistent with sensitivity analyses of the SAPHIRE model implemented in Wuhan [13]. The estimated ascertainment rates were positively correlated with the specified ascertainment rate in the initial state while the under-reported factors were negatively associated with initial ascertainment. The estimated under-reported factor on October 31 (see Table 4) decreases dramatically from 117 to 0.07 with the initial ascertainment rate increasing from 0.07 to 0.14, with an initial ascertainment rate of 0.10 providing the best fit, which is presented in this article.

The SAPHIRE model was originally developed in the context of data from China and was successfully able to delineate the transmission dynamics of COVID-19 in Wuhan [13] and in South Africa [57].

### SEIR-fansy

In the paper, we fix most parameters in our model and examine transmission dynamics only through *β* and *r*. It is necessary to design and implement a sensitivity analysis focusing on various combinations of the parameters that were previously fixed. The details of the sensitivity analyses are described in detail in [18]. The basic findings from the sensitivity analyses are summarized as follows. We observe that the predictions for the reported active cases (*P*) remains same for all parameter choices. The estimates for *R*_0_ mainly differ in the first period, although some variation is noted for the second period as well. However, the estimated *R* are almost the same for the later stages of the pandemic in the different models. For the untested cases, in some of the settings of our analysis, there are substantial deviations from the true numbers. The total number of active cases (which include both the unreported and the reported cases) also varies substantially with different parameter values. Consequently, we note how the estimation of unreported cases is sensitive to different choices for the parameter values. In particular, we see different values of *E*_0_ have the most impact on our sensitivity analysis, while different choices of *D*_*E*_ have the least impact.

The SEIR-fansy model has not been run for different countries, but it has been implemented for most Indian states separately [18] which showed that the model was able to capture the transmission dynamics of COVID-19 in most states of India quite efficiently. For instance, this model was able to match the sero-survey results of Delhi quite well [45]. For other states, the predicted reported cases came out to be quite close to the observed reported cases (with observed cases lying within the credible interval of projections).

### ICM

The parameters critical to the estimation and projection methods include the infection-to-death distribution [32], infection fatality ratio [45, 46], generation distribution [44], prior for *R*_0_ [7, 30] and seeding [7]. Researchers have performed sensitivity analysis for various choices of infection-to-death distribution and found the resultant projections to be robust under changes [7]. We used a range of values for our prior of IFR, with mean 1, 0.4 and 0.1%. We found that the model fits and estimated *R*_*t*_ are more or less the same for all three choices but certainly our estimates for total infections changes. This implies the ascertainment of cases (positive results) will be affected. Sensitivity analyses towards the choice of the generation distribution was performed by other researchers [7] who found the models to be robust against various choices. It has a very minimal effect on the estimation of time varying reproduction number and total infections by the model. We used the *R*_0_ prior suggested in both [7, 30]. We did run sensitivity on a few other choices and found that our prior choice affected the inferred *R*_*t*_ values for only the first few days and subsequent dynamics are the same irrespective of the choice. Finally, as discussed in [7] we validated our seeding scheme through an importance sampling leave-one-out cross validation scheme [58, 59].

Different versions of ICM model has been applied to 11 European countries in [7]. On a subregional basis the model is used in the USA [60], Brazil [20, 61] and Italy [21]. At a local level work the model is used for producing daily estimates for all local and regions in the UK [62, 63]. It is also used by Scotland government [64] and New York State government [65].

## Discussion

In this comparative paper we have described five different models of various stochastic structures that have been used for modeling SARS-Cov-2 disease transmission in various countries across the world. We applied them to a case-study in modeling the full disease transmission of the coronavirus in India. While simulation studies are the only gold standard way to compare the accuracy of the models, here we were uniquely poised to compare the projected case-counts and death-counts against observed data on a test period. We learned several things from these models. While the estimation of the reproduction number is relatively robust across the models, the prediction of active and cumulative number of cases and cumulative deaths show variation across models. Our findings in terms of estimates of *R*(*t*) are reflective of the national and state-level implementations of four lockdown phases [66] which are summarized in Supplementary Table S4. The largest variability across models is observed in predicting the “total” number of infections including reported and unreported cases. The degree of underreporting has been a major concern in India and other countries [67]. We note from Table 4 that the underreporting factor from SAPHIRE is much higher than those reported by SEIR-fansy and ICM. This may be attributed to the fact that SEIR-fansy and ICM both fit daily reported deaths with a pre-specified death rate (which is higher than that for unreported cases), SAPHIRE does not include daily reported death counts in the likelihood function. Additionally, SEIR-fansy also considered the false positive/negative rates of tests and the selection bias in testing, which also contribute to more accurate unreported case projections along with untested infectious case counts. With a comprehensive exposition and a single beta-testing case-study we hope this paper will be useful to understand the mathematical nuance and the differences in terms of deliverables for the models.

There are several limitations to this work. First and foremost, all model estimates are based on a scenario where we assumed no change in either interventions or behavior of people in the forecast period. This is not true as there is tremendous variation in policies across Indian states in the post lockdown phase. We did observe regional lockdowns that were enacted in the forecast period. None of our models tried to capture this variability. Second, the five models we compare are a subset of a vast amount of work that has been done in this area, including models that incorporate age-specific contact network and spatiotemporal variation [11, 68]. Third, we have not tested the models for predicting the oscillatory growth and decay behavior of the virus incidence curve, in particular, predicting the second wave. Finally, an extensive simulation study would be the best way to assess the models under different scenarios, but we have restricted our attention to India.

## Supplementary Information

**Additional file 1: Supplementary Table S1.** Summary of initial values and parameter settings for application of the SEIR-fansy model in the context of COVID-19 data from India. Unless mentioned otherwise, we use these parameter settings for all other models when applicable. **Supplementary Table S2.** Overview of projected COVID-counts for each model considered. **Supplementary Table S3.** Comparison of estimated projections and posterior estimates of model parameters across different sensitivity analysis scenarios under 21-day lockdown with moderate return, using observed data till April 14. Prior SD for R0 is 1.0. Reproduced from Ray et al., 2020 [53]. **Supplementary Table S4.** National and state-levels lockdown measures implemented over the course of COVID-19 pandemic in India. Reproduced from Salvatore et al., 2021 [66].

## Data Availability

Please visit https://github.com/umich-cphds/cov-ind-19.

## Abbreviations

ICM: Imperial College Model
MCMC: Markov Chain-Monte Carlo
MSRPE: Mean squared relative prediction error
Rel-MSPE: Relative mean squared prediction error
SEIR: Susceptible-Exposed-Infected-Removed
SIR: Susceptible-Infected-Removed
SMAPE: Symmetric mean absolute prediction error
