# Supplementary material for: A comparison of five epidemiological models for transmission of SARS-CoV-2 in India
Source: BMC Infect Dis. 2021 Jun 7;21:533. doi: 10.1186/s12879-021-06077-9 (PMC8181542; doi:10.1186/s12879-021-06077-9)
Supplement: Supplementary file 1 — Additional file 1: Supplementary Table S1. Summary of initial values and parameter settings for application of the SEIR-fansy model in the context of COVID-19 data from India. Unless mentioned otherwise, we use these parameter settings for all other models when applicable. Supplementary Table S2. Overview of projected COVID-counts for each model considered. Supplementary Table S3. Comparison of estimated projections and posterior estimates of model parameters across different sensitivity analysis scenarios under 21-day lockdown with moderate return, using observed data till April 14. Prior SD for R0 is 1.0. Reproduced from Ray et al., 2020 [53]. Supplementary Table S4. National and state-levels lockdown measures implemented over the course of COVID-19 pandemic in India. Reproduced from Salvatore et al., 2021 [66]. [file 12879_2021_6077_MOESM1_ESM.docx]

***Supplementary Table S1:*** *Summary of initial values and parameter settings for application of the SEIR-fansy model in the context of COVID-19 data from India. Unless mentioned otherwise, we use these parameter settings for all other models when applicable.*

| Parameters | Settings | Description |
| --- | --- | --- |
| $\boldsymbol{\beta}$ | Time-varying | Rate of infectious transmission by infected individuals with false negative test results. |
| $\boldsymbol{\alpha}_{\boldsymbol{P}}$ | 0.5 | Ratio of rate of spread of infection by patients who test positive, to rate of spread of infection by patients who get false negative results^a^. |
| $\boldsymbol{\alpha}_{\boldsymbol{U}}$ | 0.7 | Scaling factor for the rate of spread of infection by untested individuals^a^. |
| $\boldsymbol{D}_{\boldsymbol{e}}$ | 5.2 | Incubation period (in days). |
| $\boldsymbol{D}_{\boldsymbol{r}}$ | 17 | Recovery time (in days) for infected individuals. |
| $\boldsymbol{D}_{\boldsymbol{t}}$ | 0 | Waiting time (in days) for test result for tested individuals. |
| $\boldsymbol{\mu}_{\boldsymbol{C}}$ | 0.0562 | Death rate attributable to COVID-19^b^. |
| $\boldsymbol{\lambda, \mu}$ | $3.95\times{10}^{-5}$ | Natural birth and death rates, respectively^b^. |
| $\boldsymbol{r}$ | Time-varying | Probability of being tested for infectious individuals. |
| $\boldsymbol{f}$ | 0.30 | Probability of a false negative RT-PCR diagnostic test result. |
| $\boldsymbol{\beta}_{\boldsymbol{1}}\boldsymbol{,}\boldsymbol{\beta}_{\boldsymbol{2}}$ | 0.6 ($\beta_{1}$) and 0.7 ($\beta_{2}$) | Scaling factors for rate of recovery for undetected and false negative individuals respectively^e^. |
| $\boldsymbol{\delta}_{\boldsymbol{1}}\boldsymbol{,}\boldsymbol{\delta}_{\boldsymbol{2}}$ | 0.3 ($\delta_{1}$) and 0.7 ($\delta_{2}$) | Scaling factors for death rate for undetected and false negative individuals respectively^f^. |

1. $\alpha_{P}< 1$represents the scenario where individuals who test positive are infecting susceptible individuals are a lower rate than infected individuals with false negative test results.$\alpha_{U} < 1$ is assumed as U mostly consists of asymptomatic or mildly symptomatic cases who are known to spread the disease at a much lower rate than those with higher levels of symptoms.
2. Equal to the inverse of the average number of days for death starting from the onset of disease, times the probability of death of an infected individual. Natural birth and death rates are assumed to be equal for simplicity.
3. $\beta_{1}<1, \beta_{2}<1$ are assumed, since the recovery rate is slower for individuals with false negative test results as compared to those who have been hospitalized. The condition of untested individuals is not as severe as they consist of mostly asymptomatic people. Consequently, they are assumed to recover faster than those with positive test results.
4. $\delta_{1}<1, \delta_{2}<1$ are assumed. The death rate for those with false negative test results is assumed to be higher than those with positive test results, since the former are not receiving proper treatment. For untested individuals, the death rate is taken to be lesser because they are mostly asymptomatic. As a result, their survival probability is much higher.

**Supplementary Table S2:** Overview of projected COVID-counts for each model considered.

| Type of count projected | COVID-counts | | |
| --- | --- | --- | --- |
|  | **Cumulative**  **COVID-cases** | **Active**  **COVID-cases** | **Cumulative**  **COVID-deaths** |
| Reported | Baseline, eSIR, SAPHIRE, SEIR-fansy | eSIR, SEIR-fansy | eSIR, SEIR-fansy, ICM |
| Unreported | SAPHIRE, SEIR-fansy | SEIR-fansy | SEIR-fansy |
| Total  (reported + unreported) | SAPHIRE, SEIR-fansy, ICM | SEIR-fansy | SEIR-fansy |

| ***Supplementary Table S3:*** *Comparison of estimated projections and posterior estimates of model parameters across different sensitivity analysis scenarios under 21-day lockdown with moderate return, using observed data till April 14. Prior SD for R_0_ is 1.0. Reproduced from Ray et al., 2020 (51).* | | | | | |
| --- | --- | --- | --- | --- | --- |
| **Sensitivity Analysis** |  | **Predictions** | **Posterior Estimates** | | |
| **Scenario** | **May 1** | **May 15** | $\boldsymbol{R}_{\boldsymbol{0}}$ | $\boldsymbol{\beta}$ | $\boldsymbol{\gamma}$ |
| Under-reporting* | 25,248  [104,411] | 62,797  [343,465] | 2.28  [1.05, 4.20] | 0.20  [0.05, 0.39] | 0.09  [0.03, 0.19] |
| Case-clustering** | 24,818  [59,525] | 57,499  [189,010] | 2.81  [1.47, 4.70] | 0.16  [0.07, 0.26] | 0.06  [0.03, 0.10] |
| Prior mean for $R_{0}=2$ | 20,251  [135,034] | 42,252  [315,348] | 1.80  [0.87, 3.26] | 0.27  [0.06, 0.59] | 0.16  [0.04, 0.35] |
| Prior mean for $R_{0}=3$ | 25,757  [165,287] | 86,750  [638,770] | 2.43  [1.41, 4.07] | 0.30  [0.09, 0.60] | 0.13  [0.04, 0.30] |
| Prior mean for $R_{0}=4$ | 34,587  [213,556] | 253,935  [1,854,319] | 3.38  [2.09, 5.27] | 0.32  [0.10, 0.63] | 0.10  [0.03, 0.23] |
| * Observed case-counts are multiplied by 10, Prior mean for $R_{0}=2$  ** Assume that the cases happen in metro hotspots, use population size *N*=32 million instead of national population 1.34 billion, Prior mean for $R_{0}=2$ | | | | | |

*Supplementary Table S4: National and state-levels lockdown measures implemented over the course of COVID-19 pandemic in India. Reproduced from Salvatore et al., 2021 (66).*

| Lockdown phase | Nation-wide measures implemented | State-level variation in measures implemented |
| --- | --- | --- |
| Phase one  *(25 March – 14 April)* | All transport services – road, air and rail – were suspended, with exceptions for transportation of essential goods, fire, police and emergency services. Educational institutions, industrial establishments and hospitality services were also suspended.^a^ Services such as food shops, banks and ATMs, petrol pumps, other essentials and their manufacturing were exempted.^b^ | Gujarat, Himachal Pradesh, Karnataka, Maharashtra, Tamil Nadu, Sikkim and Telengana sealed state borders. Additionally, Maharashtra, Telengana and Tamil Nadu imposed Section 144, outlawing large gatherings of people.^c^ |
| Phase two  *(15 April – 3 May)* | Conditional relaxation promised after 20 April, subject to containment of spread. Lockdown areas classified into red, orange and green zones based on extent of spread of disease. Certain relaxations from 20 April: agricultural businesses, including dairy, aquaculture and plantations allowed to open. Cargo transportation vehicles allowed to operate. Banks and government centers distributing benefits allowed to open as well.^d^ | In interest of economic recovery, certain states like Maharashtra chose to allow specific business activities to resume, in addition to national easing of restrictions. Karnataka chose to ease the lockdown in certain areas, while Delhi, Punjab and Telengana chose to enforce strict lockdown measures.^e^ |
| Phase three  *(4 May – 17 May)* | Zonal classification of regions into red, orange and green zones continued, with normal movement allowed in green zones. Movement of private and hired vehicles allowed in orange zones and red zones remained in lockdown. Zonal classifications revised on a weekly basis.^f^ | Delhi allowed public- and private-sector offices to reopen, with social distancing measures in place. Maharashtra eased most industrial and commercial activities. Gujarat, and. Jharkhand allowed no relaxation, while Bihar, Uttar Pradesh, Rajasthan and Madhya Pradesh chose to mostly adhere to guidelines issued by the Union Home Ministry.^g^ |
| Phase four  *(18 May – 31 May)* | Unlike the previous phases, states were given a larger say in the demarcation of green, orange and red zones and the implementation roadmap. Red zones were further divided into containment and buffer zones. Local administrative bodies were given the authority to demarcate containment and buffer zones.^h^ | Restricted individual movement allowed in Delhi, while Maharashtra, Tamil Nadu and Telengana extended the lockdown further. Karnataka allowed public transport with social distancing measures, while West Bengal began easing workplace restrictions. Standalone shops were allowed to open for short durations.^i^ |

1. Guidelines on measures to be undertaken by ministries/departments of Government of India, State/Union Territory Governments and State/Union Territory Authorities for containment of COVID-19 epidemic in the Country (https://www.mha.gov.in/sites/default/files/Guidelines.pdf)
2. The Economic Times: India's 21-day lockdown to counter coronavirus: What's exempt, what's not, *25 March 2020* (https://economictimes.indiatimes.com/news/politics-and-nation/india-21-day-lockdown-what-is-exempted-what-is-not/articleshow/74798725.cms)
3. Wikipedia <https://en.wikipedia.org/wiki/Indian_state_government_responses_to_the_COVID-19_pandemic>
4. BBC: Coronavirus lockdown guidelines: What has India changed under new rules? *April 15, 2020 (*[*https://www.bbc.com/news/world-asia-india-52290761*](https://www.bbc.com/news/world-asia-india-52290761)*)*
5. Hindustan Times: Complete list of states with no relaxation in lockdown 2.0 restrictions *20 April 2020* (https://www.hindustantimes.com/india-news/complete-list-of-states-with-no-covid-19-lockdown-2-0-relaxation/story-pfE5K3Pn5LSZrgFEvC84hO.html)
6. India Today: Full list of Red, Yellow, Green Zone districts for Lockdown 3.0, *1 May 2020* (https://www.indiatoday.in/india/story/red-orange-green-zones-full-current-update-list-districts-states-india-coronavirus-1673358-2020-05-01)
7. Hindustan Times: Covid-19 lockdown 3.0: A look at relaxations, restrictions across major states in India, *4 May 2020* (https://www.hindustantimes.com/india-news/coronavirus-update-covid-19-lockdown-3-0-a-look-at-relaxations-restrictions-across-major-states-in-india/story-J5Z2IypwiagUTFf1wYW0jN.html)
8. The Economic Times: Lockdown 4.0 guidelines: Nationwide lockdown extended till May 31, with considerable relaxations, *21 May 2020* (https://economictimes.indiatimes.com/news/politics-and-nation/centre-extends-nationwide-lockdown-till-may-31-with-considerable-relaxations/articleshow/75790821.cms)
9. BBC: India lockdown 4.0: What is allowed in your city? *19 May 2020* (https://www.bbc.com/news/world-asia-india-52707371)
